# Aspirin inhibits rotavirus replication and alters rat gut microbial composition

**DOI:** 10.1186/s12985-023-02199-5

**Published:** 2023-10-17

**Authors:** Wei Zhao, ZhouPing Li, Mei Ling Yu, Yang Liu, Chang Cheng Liu, Xue Jiao Jia, Meng Qi Liu, Yong Gang Li

**Affiliations:** 1 College of Basic Medical Sciences, Jinzhou Medical University, Jinzhou, China; 2 The first affiliated hospital of Jinzhou Medical University, Jinzhou, China

**Keywords:** Rotavirus, Aspirin, Virus replication, Gut microbiota, 16S rRNA gene sequencing, Rat model

## Abstract

**Background:**

Aspirin is widely used to treat various clinical symptoms. Evidence suggests that aspirin has antiviral properties, but little is known about its specific effect against rotavirus.

**Methods:**

MA104, Caco-2, and CV-1 cells were infected with rotavirus, and aspirin was added after 12 h. Viral mRNA and titer levels were measured by qRT-PCR and immunofluorescence assays. For in vivo validation, forty specific-pathogen-free SD rats were randomly divided into oral aspirin (ASP) groups and control (NC) groups. 16 S rRNA gene sequencing was performed to identify gut microbiota. After 6 months of continuous ASP/NC administration, the rats were infected with rotavirus. Fecal samples were collected over a 30-day time course, and viral levels were quantified. Proinflammatory cytokines/chemokine levels were measured by ELISA.

**Results:**

Aspirin inhibited rotavirus infection in cell lines and in rats. The effects of aspirin on viral replication were associated with the alteration of gut microbiota composition by aspirin, including increased abundance of Firmicutes and decreased abundance of Bacteroidetes after aspirin treatment. Mechanistically, aspirin reduced IL-2 and IL-10 levels, and increased IRF-1 and COX-2 levels. Aspirin blocked rotavirus replication in vitro and in vivo, which might be related to effects on IRF-1, COX-2, chemokines, and gut microbial composition.

**Conclusion:**

These results indicate that long-term oral aspirin administration reduces rotavirus infection. Intestinal virus infection may be suppressed in elderly patients who take aspirin for a long time. The change of their Gut microbiota may lead to functional disorder of the intestinal tract, which may provide some reference for clinical adjuvant probiotics treatment.

**Supplementary Information:**

The online version contains supplementary material available at 10.1186/s12985-023-02199-5.

## Background

Rotavirus is a non-enveloped, double-stranded RNA virus of the Reoviridae family that is the most important cause of severe viral gastroenteritis and may be life-threatening [1, 2]. Nearly every child worldwide under age 5, elderly, and chronic disease patients are the main groups of rotavirus infections [3]. Rotavirus generally replicates in mature small intestine enterocytes and enters the bloodstream, resulting in antigenemia and viremia [4, 5]. Despite extensive experimental research on the epidemiology of rotavirus [6, 7], there is a lack of in-depth studies on rotavirus infection in elderly or chronic disease patients who have been taking medication for a long time in clinical practice.

Aspirin, also called acetylsalicylic acid, is commonly used to relieve minor aches and pains [8], reduce fever [9], decrease inflammation [10], and prevent platelet aggregation by blocking the formation of thromboxane A2 [11]. The ability of aspirin to inhibit blood clots underlies its utility as a preventative for heart attacks and strokes [12–14]. Low-dose aspirin regimens (75–100 mg/day) can effectively suppress platelet aggregation without affecting important endothelial cell functions [13]. Reports have also shown that soluble aspirin given by mouth in divided dosage significantly decreases intestinal fluid loss and diarrhea in infants and young children with acute gastroenteritis [15]. Though limited information is available regarding the potential effect of aspirin against enteric infectious agents, such as viral pathogens that cause gastroenteritis, it has been shown to function as an antiviral against a panel of human pathogenic viruses, including influenza A virus [16, 17], human rhinovirus (HRV) [18], varicella zoster virus (VZV) [19], cytomegalovirus(CMV) [20], hepatitis C virus (HCV) [21, 22], Epstein Barr virus (EBV) [23], human respiratory syncytial virus (RSV) [24], Japanese encephalitis virus (JEV) [25], dengue virus (DENV) [26] and human immunodeficiency virus (HIV) [27, 28].

Most recently, a potential therapeutic role for aspirin in treating COVID-19 has been advocated due to its anti-inflammatory, antiplatelet aggregation, and anticoagulant effects, as well as its modulation of the immune system and possible inhibition of viral replication or entry [29–31]. However, whether aspirin can inhibit rotavirus replication and infection in vitro and in vivo has not been reported.

To investigate the antiviral effect of aspirin in *vitro* and its long-term effect on chronic rotavirus infection, we used an in-cell model to evaluate the antiviral effect of aspirin in *vitro.* A long-term aspirin administration drug model in rats was established and further infected with rotavirus. The effect on rotavirus infection was evaluated in both in vitro and in vivo models. The results indicated that aspirin treatment could reduce rotavirus replication in cells, and rotavirus infection was also inhibited in rat models. The gut microbiota of rats taking aspirin for a long time was changed specifically, which probably led to the difference in rotavirus infection with or without aspirin orally taken in rats. In addition, aspirin could increase the expression of IRF-1 and COX-2 while inhibiting the expression of IL-2 and IL-10. These results reference the clinical medication and probiotic use for older adults who have been taking aspirin for a long time when infected with rotavirus.

## Materials and methods

### Viruses, cell lines, and viral infection

Rotavirus SA11 strain (The simian RV SA11 strain, G3P) was provided by Dr Kobayashi, Osaka University, Japan. Fetal African green monkey kidney CV-1 cells, rhesus monkey kidney MA104 cells, and human colon adenocarcinoma Caco-2 cells (Cell Resource Center, IBMS, and CAMS/PUMC, Beijing, China) were grown in minimal-essential medium (Thermo Fisher Biochemical, Beijing, China) supplemented with 10% fetal bovine serum (GIBCO, Paisley, UK) and antibiotics (Sigma-Aldrich, St. Louis, MO, USA). MA104 cells were used to propagate rotavirus SA11. Caco-2 and CV-1 cells were infected as previously described [32]. Briefly, cell monolayers were washed three times with phosphate-buffered saline (PBS) and inoculated with viral stocks after treatment with 1 µg/µl of trypsin (Sigma-Aldrich, St. Louis, MO, USA) for 60 min at 37ฒC. After 1 h adsorption, the cells were plated in a Minimal-Essential Medium containing 0.5 µg /ml trypsin. The virus titer was counted by fluorescent focus assay, and the RNA viral load was quantified by qRT-PCR, as described [33].

### Determination of the aspirin cytotoxicity

To identify nontoxic doses of aspirin for use in the assessment of antiviral activity, we continuously cultivated cells with serial dilutions of aspirin. Cells without aspirin treatment were used as controls. CCK8 assays were performed after 12, 24, 36, and 48 h, and the cytotoxicity thresholds were determined. Cells were plated in 96-well plates at a density of 5000 cells in 100 µL medium per well 1 day before the experiment. To determine the limits of toxic concentrations, cells were cultivated together with varying dilutions (ranging from 10 to 0.005 mol/ml ) of aspirin and the control at 37 °C and 5% CO2. The cell viability was examined by CCK-8 kit (Benefiting Lives, Serving Life Science, Beijing, China) according to the manufacturer’s instruction.

### Experimental design for oral aspirin treatment of rats in a rotavirus infection model

Forty 1-month-old specific-pathogen-free SD rats (average weight 200 g, male/ female) were provided by the Laboratory Animal Center at Jinzhou Medical University (Liaoning Province, China). All experimental rats were housed in individually ventilated autoclaved cages in the same specific-pathogen-free room. The living environment, feeding conditions, and microbial conditions of the animals were consistently maintained on a 12-h light/dark cycle at 22 ± 2 °C with 40–70% humidity. The rats were randomly divided into the oral aspirin (ASP) group and the control (NC) group. The ASP group was orally administered aspirin (aspirin Enteric-coated Tablets 100 mg/Tablet, Bayer AG, Germany) dissolved in 0.5% carboxymethyl cellulose solution according to the following calculation: Rat dose administered =(Human dose, mg/kg) × 70 kg× 0.018/200 g = 630 mg per rat (3.15 mg/ gram of rat body weight. The NC group was orally administered 1 ml of 0.5% carboxymethyl cellulose solution as a control. The aspirin or control solution was administered continuously for 6 months (a flow diagram was shown in supplement Fig. 1).

**Fig. 1:**
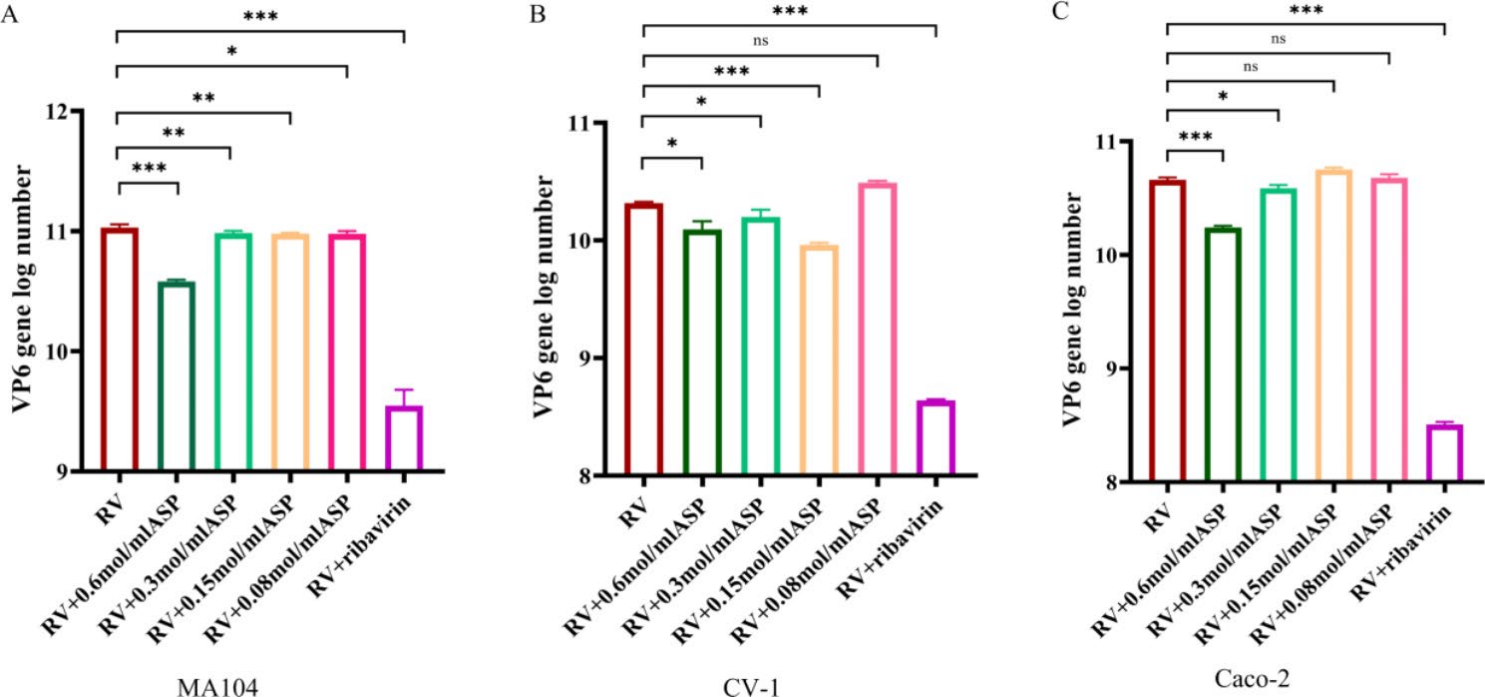
qRT-PCR assay of the dose-dependent antiviral activity of aspirin against rotavirus Cells were infected with the rotavirus-SA11 strain, and then different concentrations of aspirin (0.6, 0.3, 0.15, and 0.08 mol/ml) were added to the culture medium for 12 h. Levels of expression of the rotavirus VP6 gene were determined by qRT-PCR assay. The percentage inhibition in the cell cultures (relative to infected but non-treated cells) is shown for MA104 (**A**), Caco-2 (**B**), and CV-1 (**C**) cells. Ribavirin (0.2 mol/ml) was included as an internal positive control. Data were derived from three replicates and are representative of three independent experiments. All viruses were used with 107 FFU/ml. RV, rotavirus; ASP, aspirin. * p < 0.05; ** p < 0.01; *** p < 0.001

After 6 months, the two groups were given the same amount of rotavirus SA11 (10^6^ PFU/ml per day) for 30 days of persistent infection. The body weights were recorded throughout the intervention. Fecal samples were detected at day 6, 12, 18, 24, and 30 days post-infection. Viral titers and virus gene replication were quantified by fluorescent focus assay and qRT-PCR, respectively. The colon and rectum mucosa of rats were collected using autoclaved operating knife blades and stored in sterile tubes at − 80 °C.

All animal experiments were performed following the Jinzhou Medical University guidelines and approved by the Animal Welfare and Ethical Review Board at Jinzhou Medical University (approval ID: 202,014). All animal infections and infectious work were performed in Biosafety Level 2 facilities.

### 16 S rDNA sequencing of gut microbiota in rat stool samples

To assess microbiota composition, the stool samples of 16 rats (8 from the ASP group and 8 from the NC group) were evaluated by 16 S rRNA gene sequencing. DNA was extracted from rat fecal pellets using the QIAamp DNA Stool Mini kit (Qiagen Inc., Valencia, CA, USA) according to the manufacturer protocols. The V3–V4 region of the bacterial 16 S ribosomal RNA genes (342 F and 806R) was amplified by qRT-PCR as previously described [34]. The samples were sequenced on an IlluminaMiSeq platform (Illumina, Inc., San Diego, CA, USA), and the reads were assembled for subsequent 16 S analysis.

Alpha diversity was calculated using software to normalize the number of clean reads in each sample [35]. Beta diversity shared between the two groups was calculated using UniFrac distance matrices [36]. A principal coordinate analysis plot of group similarities was based on the UniFrac distance. Linear discriminant effect size (LEfSe) analysis was used to determine the features most likely to explain differences between the ASP and NC groups. Different features with an LDA score were identified [37]. A statistical analysis of the taxonomic and functional profiles was used for further exploration by second-level KEGG analysis using Reconstruction of Unobserved States (PICRUSt) [38, 39].

### qRT-PCRof COX-2 and IRF-1 mRNA

Total RNA was extracted from Caco-2 cells using TRIzol reagent (Invitrogen), according to the manufacturer’s instructions. Next, cDNA was reverse-transcribed from 1 µg total RNA using PrimeScript Reverse Transcriptase (TaKaRa Bio, Dalian, China).qRT-PCR was performed using the SYBR PrimeScript RT-kit (TaKaRa Bio, Dalian, China) and the following primers: COX-2: Forward 5′-CGGTGAAACTCTGGCTAGACAG-3′, Reverse 5′-GCAAACCGTAGATGCTCAGGGA-3′, IRF-1: Forward 5’-ACCCTGGCTAGAGATGCAGA-3’,

Reverse 5’-GCTTTGTATCGGCCTGTGTG-3’; GAPDH: Forward 5-GTCT CCT CTGA CTT CAACAGCG-3, Reverse 5’-ACC ACCC TGTT GCT GTA GCC AA-3’.

Expression of COX-2, IRF-1, and GAPDH transcripts were analyzed using the Threshold cycle (CT) average value of triplicates method. The relative expression of viral RNA and GAPDH was calculated using the 2-^ΔΔ^CT method. Results were analyzed using Bio-Rad IQTM5 optical system software. Differences between groups were examined using Student’s t-test, with P < 0.05 as the threshold for statistical significance. Data are presented as the mean ± SD.

### Enzyme-linked immunosorbent assay (ELISA)

The rat colon tissues were removed and slit open longitudinally along the main axis, then washed with normal saline (0.9% NaCl). The internal mucosa of the colon was gently scraped off with autoclaved operating knife blades and placed in phosphate-buffered saline for vortex mixing. The supernatant was for ELISA detection after centrifugation. ELISA kits (Jiangsu Meimian industrial Co., Ltd) were used to detect prostaglandin E2 (PGE2) (No. 875), adenosine triphosphate (ATP) (No. 20,973), thromboxane A2 (TXA2) (No. 7477), and cAMP (No.14,171) in serum, and TNF-α (No. 1113) in colon mucosa of each of the groups in rat model. IL-10(ZK-4375) and IL-2 (ZK-5859) in colon mucosa were also detected using ELISA kits (*Shanghai Zhen Ke Biological Techology Co., Ltd.)*

### Statistical analysis

Statistical analyses were performed using GraphPad Prism Version 5.01 (La Jolla, CA, USA). Statistical comparisons of the experimental data between the treatment and control groups were performed using a two-tailed Student’s t-test or one-way analysis of variance (ANOVA). Experimental results were expressed as the mean ± standard deviation (SD). P values < 0.05 were considered statistically significant.

## Results

### Aspirin inhibits in vitro rotavirus replication

Before the assays determining the antiviral activity of aspirin, its cytotoxic effect on cells used for the antiviral assays had to be determined. Concentrations of 10 mol/ml aspirin resulted in lysis of the cell monolayers to different degrees and > 20% cytotoxicity for all three cell lines. However, cytotoxicity of < 20% was achieved within the 2.5 mol/ml to 0.3 mol/ml range for MA014 cells (Supplementary Fig. 1A), and the 1.3 mol/ml to 0.08 mol/ml range for Caco-2 (Supplementary Fig. 1B) and CV-1 cells (Supplementary Fig. 1C). No toxic effects of aspirin were detectable at concentrations < 0.6 mol/ml for MA104 and CV-1 cells and < 0.08 mol/ml for Caco-2 cells. Therefore, we used an upper limit of 0.6 mol/ml aspirin in subsequent assays of antiviral activity.

**Fig. 2:**
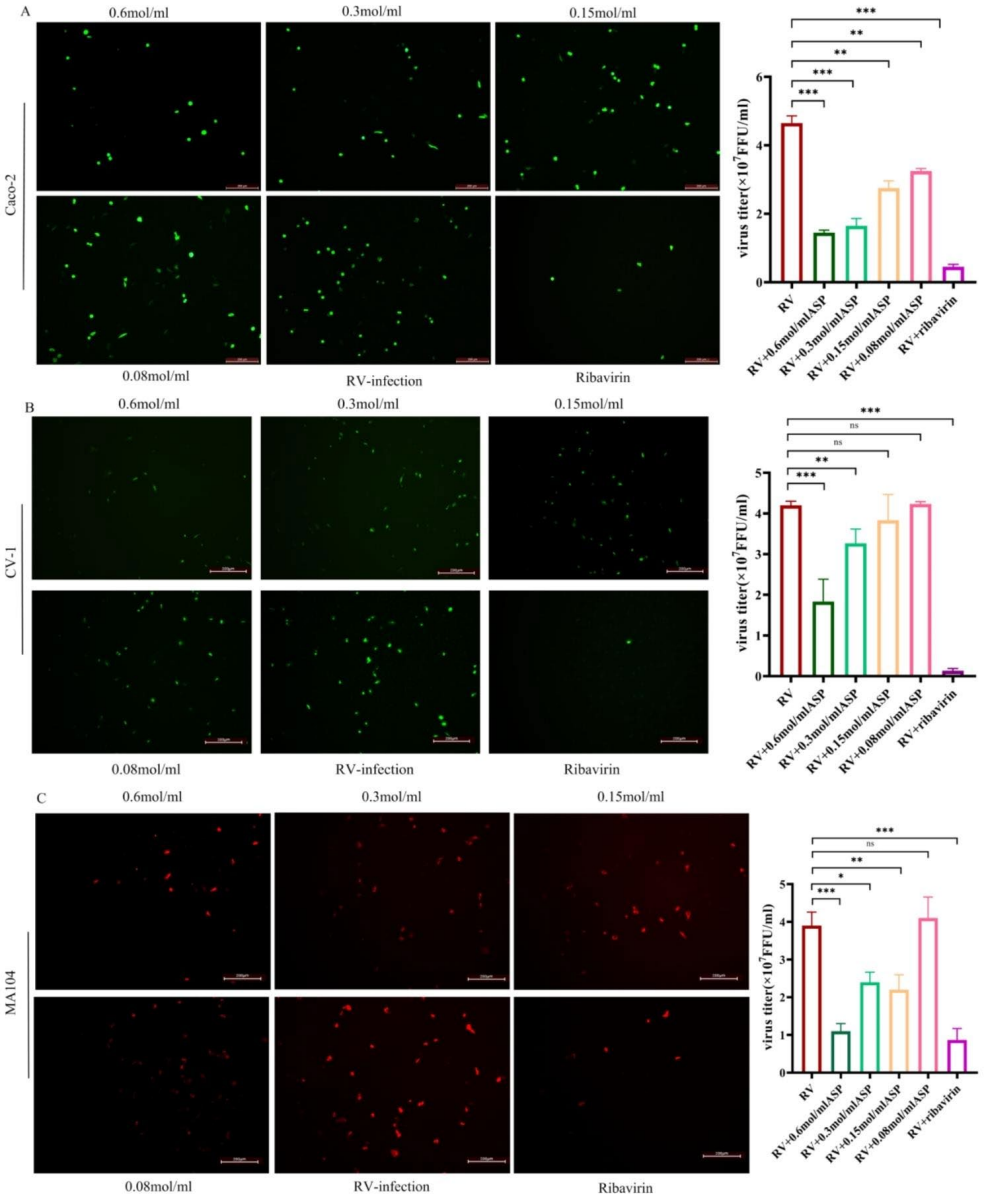
Immunofluorescent assay of the antiviral activity of aspirin against rotavirus Fluorescent focus assays were performed for quantification of the antiviral activity against rotavirus in different host cells. (**A**), Caco-2 (**B**), CV-1 (**C**) MA104. Different concentrations of aspirin were added in the culture medium after virus infection (10^7^ FFU/ml). After 12 h, supernatants were collected, and virus titers were determined by the immunofluorescence method. Non-treated virus-infected cells were used as a negative control, and ribavirin (0.2 mol/ml) as an internal positive control. Virus titer= (Fluorescence quantity × Dilution ratio)/volume of virus added per well × 1000. RV, rotavirus; ASP, aspirin. *P = 0.0003, **P = 0.0002, ***P = 0.0001

To determine whether aspirin could inhibit rotavirus replication, we infected MA104, Caco-2, and CV-1 cells with the rotavirus SA11 strain and then added different concentrations of aspirin to the culture medium. After 12 h, rotavirus loads were determined by qRT-PCR of the VP6 gene. Antiviral effects of aspirin were observed in all three cell lines (Fig. 2). In MA104 (Fig. 2A) and CV-1 (Fig. 2B), 0.6 mol/ml aspirin induced ~ 4.5% inhibition of viral replication, and 0.3 and 0.15 mol/ml dilutions also harmed virus replication. Furthermore, in Caco-2 cells (Fig. 2C), 0.6 mol/ml aspirin induced ~ 6.5% inhibition of viral replication, and 0.3 mol/ml also had an effect, whereas 0.15 mol/ml had no detectable effect on virus replication. These results suggested that the effect of aspirin was dose-dependent and cell-dependent but that 0.6 mol/ml aspirin was effective at inhibiting rotavirus replication in all three cell lines.

**Fig. 3:**
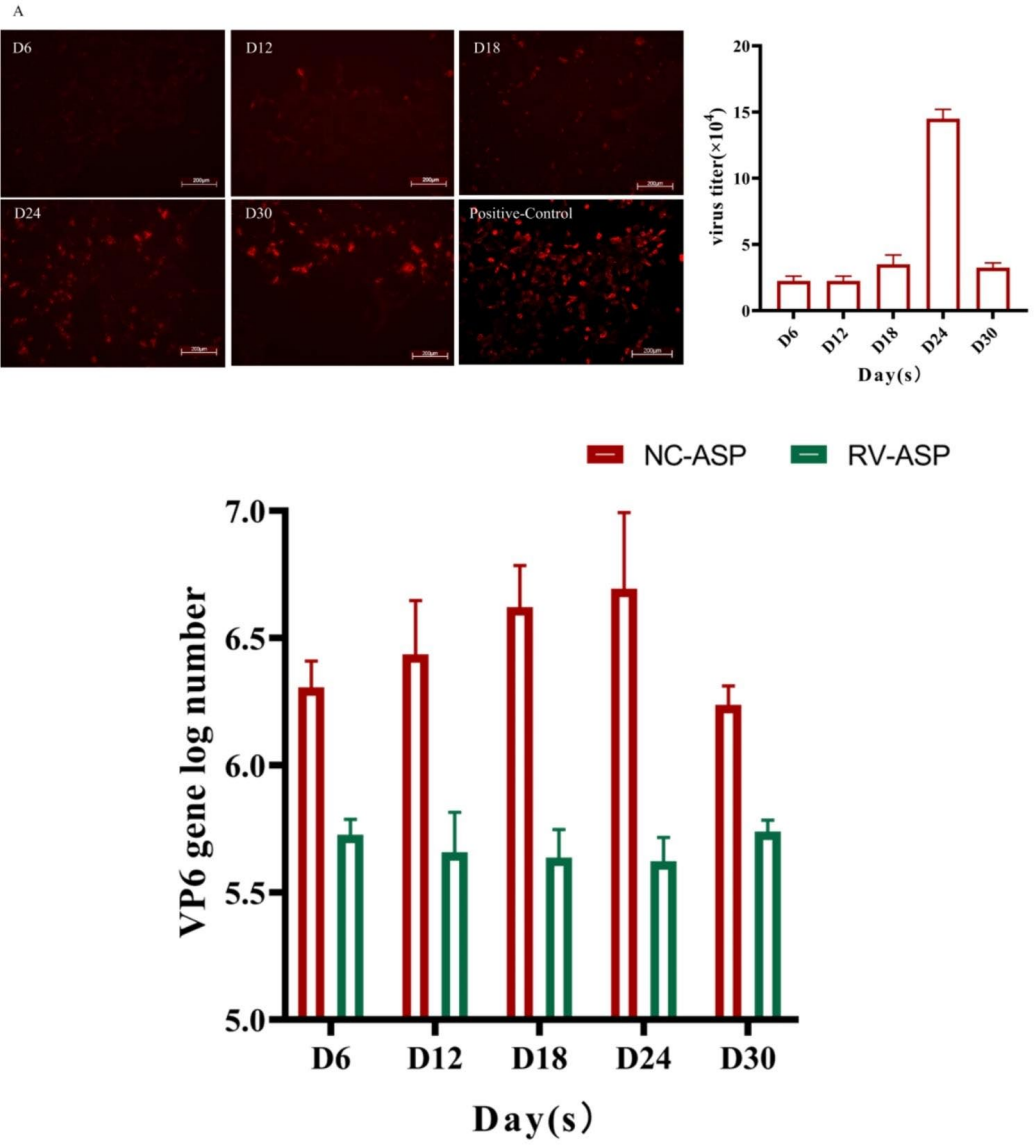
Determination of the antiviral activity of long-term aspirin administration in the rotavirus-infected rat model Forty rats were randomly distributed into the control (NC) (oral 0.5% carboxylmethylcellulose solution 1 ml) and ASP group (oral aspirin). After 6 months, the two groups were infected with the same amount of SA11 (10^6^ PFU/ml per day) for 30 days (NC-RV and ASP-RV). The viral titer and virus RNA in rat fecal were evaluated on 6, 12, 18, 24, 30 days by fluorescent assay (**A**) and qRT-PCR (**B**). RV, rotavirus; ASP, aspirin

To verify that aspirin inhibited rotavirus replication, we performed immunofluorescence assays (Fig. 3). The results confirmed that aspirin inhibited the viral titer in a dose-dependent manner, with the most pronounced inhibitory effect observed at 0.6 mol/ml and 0.3 mol/ml in all three host cell lines. These results confirmed that 0.6 mol/ml aspirin most effectively inhibited rotavirus infection and replication in host cells, thus confirming its potential utility as an antiviral agent.

### Long-term aspirin administration promoted antiviral activity in the rat rotavirus infection model

Given the above evidence for aspirin’s ability to inhibit rotavirus replication in *vitro*, we sought to evaluate whether Long-term aspirin administration inhibited rotavirus persistent infection in vivo. Obvious diarrhea symptoms were not observed, and neither the aspirin nor the viral infection influenced body weight and the growth of the rats (data not shown). According to immunofluorescence foci (FFU/mL) results, the number of fluorescent cells was the highest on day 24 and decreased on day 30 in the NC-RV group (Fig. 3A); in contrast, fluorescent cells could not be detected in the ASP-RV group at any stage of the 30-day time course(Supplementary Fig. 2).Consistent with the qRT-PCR results, the ASP-RV group of rats had a lower viral copy number throughout the time course than only RV-infected rat (Fig. 3B). These results suggested that long-term oral aspirin administration in rats reduced rotavirus infection.

### Long-term aspirin administration aspirin altered the gut microbiota in rats

**Fig. 4:**
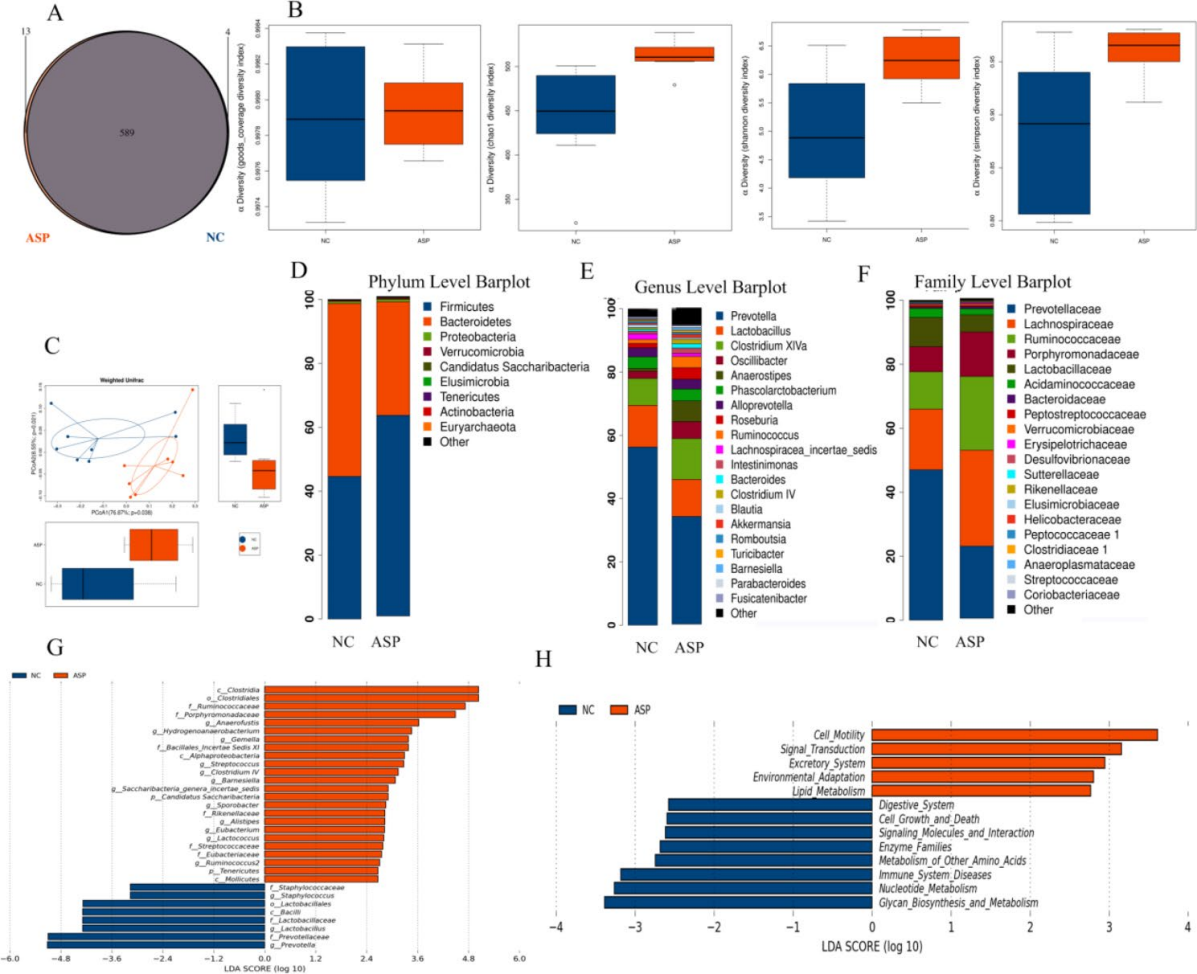
Characterization of gut microbial alterations in the oral aspirin rat model (**A**) Venn diagram of OTUs in the aspirin (ASP) and control (NC) groups. (**B**) The bacterial diversity in the ASP and NC groups was estimated by the observed-species diversity, Chao-1, Shannon index and Simpson indexes. (**C**) The beta diversity in the NC and ASP groups was assessed by Weighted Unifrac ANOSIM analysis. A principal coordinate analysis plot of the similarities between the groups was based on the UniFrac distances. Principal components PCOA1 and PCOA2 explained 76.87% and 8.55% of the variance, respectively. (**D**-**F**) Relative abundances of OTUs assigned at the Phylum, Genus and Family levels. (**G**) LEfSe analysis of taxonomic differences among the NC and ASP groups. Taxonomic biomarkers found in the ASP (red) and control (blue) groups were identified by linear discriminant analysis effect size (LEfSE). The LDA scores obtained from the microbial groups with significant differences in the ASP and NC groups were confirmed by regression analysis (LDA threshold 2). (**H**) Functional differences in predicted genes in 16SrRNA gene sequencing related to KEGG pathways at level 2

To investigate gut microbial alterations during oral aspirin administration, we randomly selected 8 rats each from the NC and ASP groups and performed 16 S rRNA gene sequencing. As seen in the Venn diagram (Fig. 4A), 589 operational taxonomic units (OTUs) were shared by ASP and NC group. As shown in Fig. 4B, the gut microbiota richness and community diversity were markedly lower in the NC group than in the ASP group, indicating that more bacterial species were colonized in the rats after treatment with aspirin (Fig. 4B). Consistent with β-diversity analysis, the gut microbiota in the NC group differed from that in the ASP groups. The results suggested that the ASP and NC groups had a clear separation and distinct differences in the bacterial composition (Fig. 4C). We next examined differences in bacterial taxa among the two groups at the phylum, genus, and family levels. At the phylum level, the relative abundance of Firmicutes was lower in the NC group compared to the ASP group. In contrast, Bacteroidetes was higher in the NC group compared to the ASP group. Consequently, the Firmicutes/Bacteroidetes ratio was lower in the NC group than in the ASP group, indicating that the gut microbial composition and structure had significantly changed after oral aspirin treatment (Fig. 4D). At the genus level, Prevotella and Lactobacillus were abundant in the NC group but decreased in ASP groups, whereas Anaerostipes ,ClostridiumXlVa, and Alloprevotella were enriched in the ASP group (Fig. 4E). At the family level, Prevotellaceae and Lactobacillaceae were enriched in the NC group but decreased in ASP groups, while Lachnospiraceae, Ruminococcaceae, and Porphyromonadaceae were enriched in the ASP group (Fig. 4F).

To identify salient features among the two groups, we performed linear discriminant analysis effect size (LEfSe; LDA Effect Size) analysis. Prevotella, Lactobacillus, and Staphylococcus were enriched in the NC group; while Anaerofustis, Gemella, and Streptococcus were the predominant genera in the ASP group (Fig. 4G). To evaluate the functional consequences of these differences, we performed second-level KEGG pathway analysis. Functions related to “Digestive System”, “Cell-Growth and Death”, “Signaling Molecules and Interaction”, “Immune System Diseases” and “Nucleotide Metabolism” were enriched in the NC group, while functions related to “Cell Motility”, “Signal Transduction”, “Excretory System”, “Environmental Adaptation” and “Lipid Metabolism” were enriched in the oral aspirin rat model (Fig. 4H). These results suggested that differences in the gut microbiota might contribute to the differential response to rotavirus in rats treated with aspirin.

### Long-term aspirin administration lowered the expression levels of proinflammatory cytokines/ chemokines in rats

**Fig. 5:**
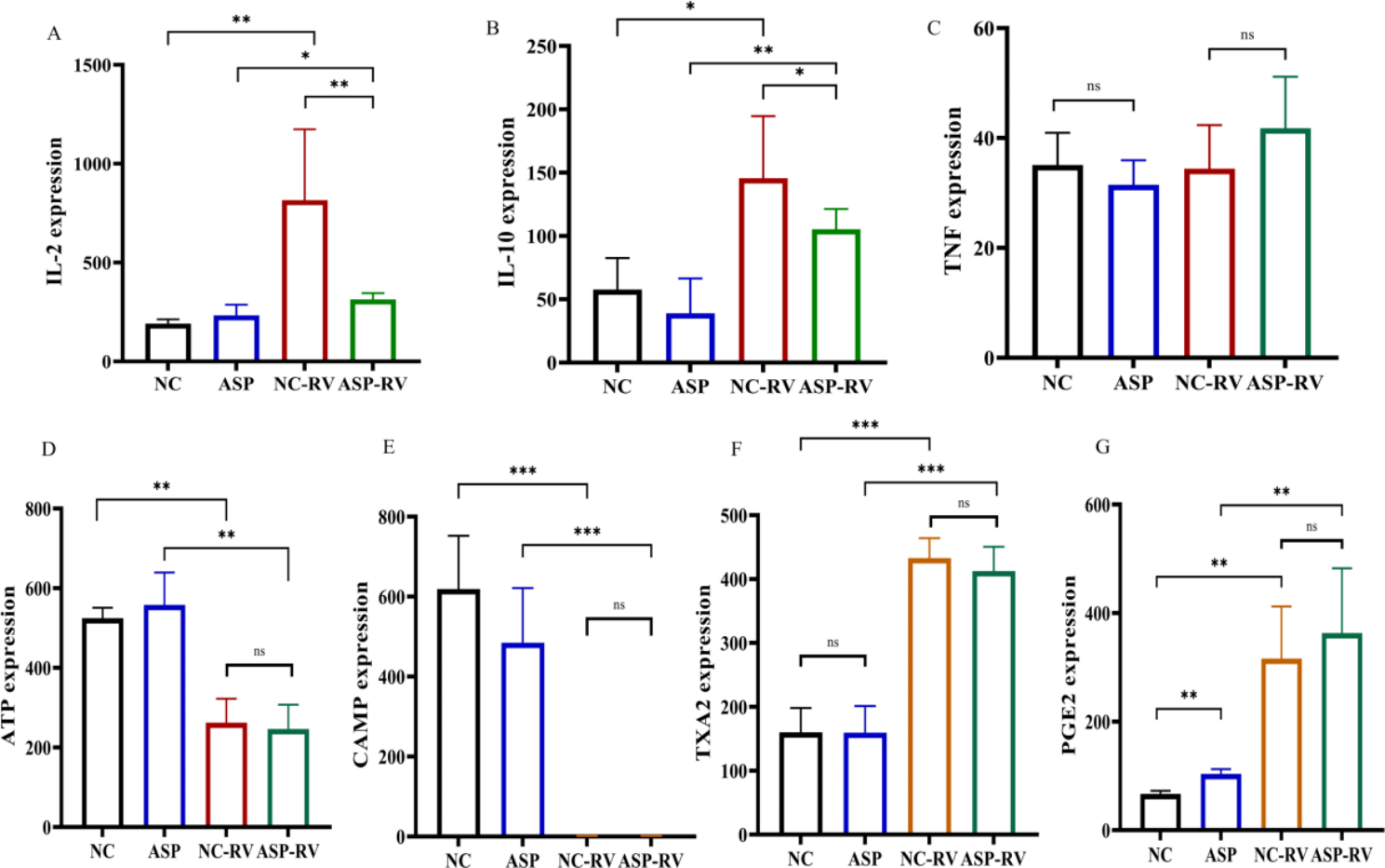
Proinflammatory cytokine/ chemokine levels in the oral aspirin rat rotavirus infection model The levels of pro-inflammatory cytokines/chemokines in control rats (NC) and rats treated with aspirin (ASP) continuously for 6 months and/or infected with SA11 rotavirus (RV) for 30 days were detected by ELISA. (**A**) IL-2, (**B**) IL-10, (**C**) TNF, (**D**) ATP, (**E**) cAMP, (**F**) TXA2, (**G**) PGE2.* p < 0.05; ** p < 0.01; *** p < 0.001

Rotavirus-infected rats exhibited higher levels of interleukin-2 (IL-2) (Fig. 5A), and interleukin-10 (IL-10) (Fig. 5B), and the increased levels were partially reversed when aspirin was administered in conjunction with infection. On the other hand, the levels of tumor necrosis factor-α (TNF-α) were unchanged (Fig. 5C), and the serum levels of ATP and cAMP were decreased by rotavirus. However, only cAMP was affected by aspirin (Fig. 5D, E). Conversely, the levels of TXA2 and PGE2 were increased by rotavirus, and the levels of PGE2, but not TXA2, were further increased by aspirin treatment (Fig. 5F, G).

**Fig. 6:**
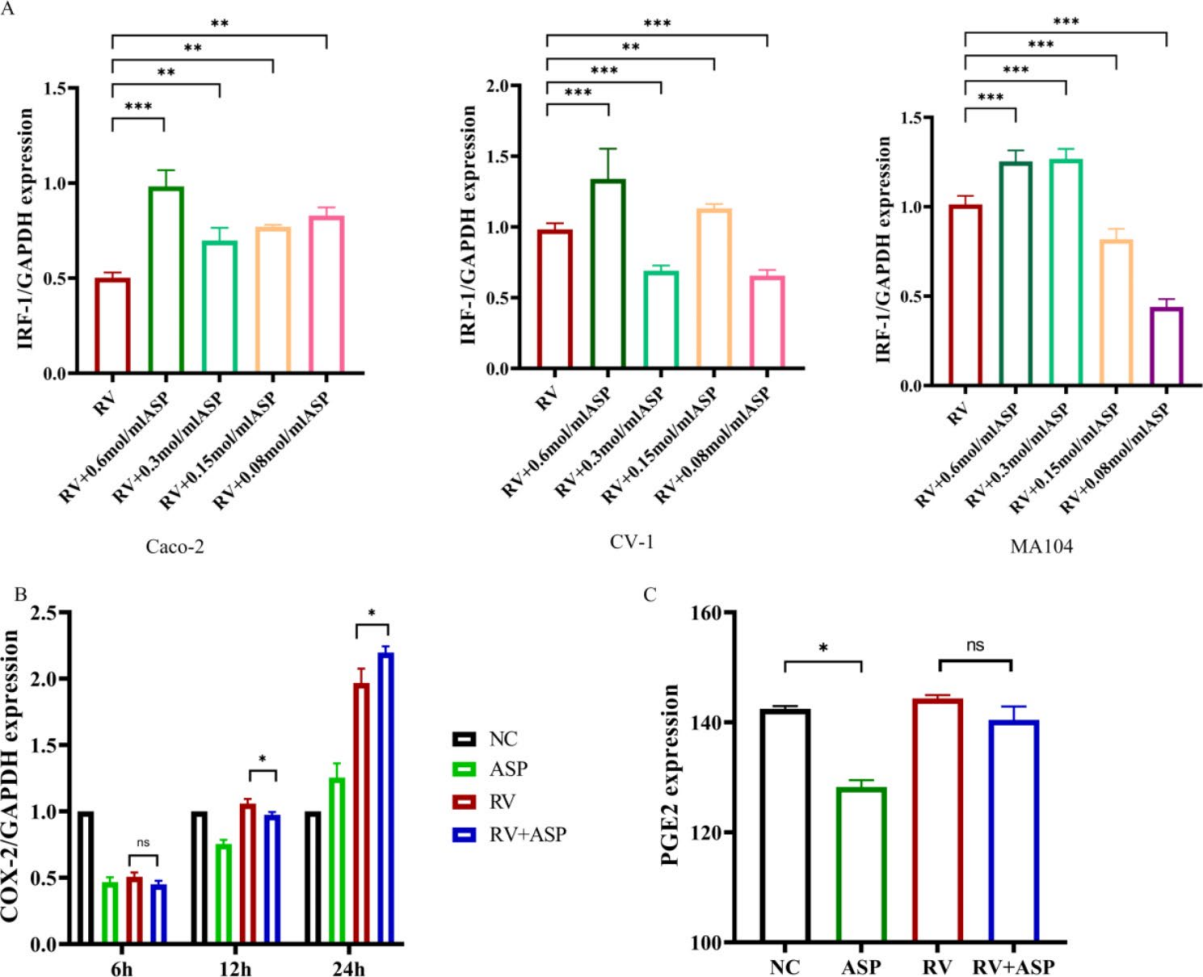
Aspirin promotes the expression of IRF-1, COX2 and PGE2 Caco-2, CV-1 and MA104 cells were infected with rotavirus (10^7^ FFU/ml) and 0.6, 0.3, 0.15, and 0.08 mol/ml of aspirin were added for 12 h. qRT-PCR was performed to analyses IRF-1 mRNA expression in each of the three host cells (**A**). Data are presented as the average ± SD. ***P < 0.0001. (**B**) The levels of COX-2 mRNA expression were determined by qRT-PCR using COX-2-specific primers in infected cells relative to noninfected cells at 12 h. Error bars indicate the standard errors of the means (n = 3). (**C**) Secreted PGE2 in the media was quantified using the ELISA method. Significant differences (*, P 0.01;) between samples are indicated. Error bars indicate the standard errors of the means (n = 3)

To evaluate the mechanism of aspirin in inhibiting rotavirus, we detected its potential effect on IRF-1, a transcriptional activator of interferon, an antiviral host protein [40]. The results show that 0.6 mol/ml aspirin induces IRF-1 mRNA in all three host cells (Fig. 6A), which raises the possibility that interferon may account in part for the antiviral activity of aspirin.

Previous studies have shown that rotavirus infection of Caco-2 cells increases the expression of COX-2 mRNA and the secretion of PGE2 prostaglandin [41].As a key feature of its mechanism, aspirin inhibits prostaglandin synthesis by irreversibly inactivating cyclo-oxygenase 1 and 2 (COX-1 and COX-2) [42]; Therefore, we evaluated the effects of aspirin on COX-2 and PGE2 in host cells. At 24 h post-infection, the levels of COX-2 mRNA expression were increased by rotavirus infection (RV group); however, the addition of aspirin in rotavirus-infected cells (RV + ASP), COX-2 mRNA expression was not significantly changed (Fig. 6B). Although the mRNA expression level of PGE2 decreased in the host cell after adding aspirin, there was no statistical significance between the rotavirus infection group and the RV + ASP group. These results indicated that COX and PGE2 comprised important early mediators of rotavirus infection, but aspirin treatment might not block COX mRNA 2 and PGE2 activity to inhibit rotavirus infection in early mediators.

## Discussion

Aspirin has been used for at least 115 years to relieve minor aches, pains, fever, and inflammation [18, 43]. Additionally, aspirin inhibits platelet aggregation via blockade of the formation of thromboxane A2 in platelets, making it particularly useful as a preventative for heart attacks and strokes [12, 44, 45]. More recently, accumulating data demonstrate the relationship between aspirin and infectious diseases caused by bacteria, viruses, fungi, parasites, and their associated infections [46]. However, only some research studies have examined the ability of aspirin to reduce viral replication.

In this study, we evaluated the potential of aspirin to inhibit rotavirus infection in cells. The results showed that 0.6 mol/ml aspirin inhibited rotavirus replication in three cell lines (MA101, CV-1, and Caco2). Several scientific studies supported the hypothesis that aberrant induction of COX-2 and the prostaglandin cascade played a significant role in viral-mediated cellular damage [49, 50]. Rotavirus infection was attenuated by a nonspecific COX inhibitor, and a COX-1/COX-2-specific inhibitor [47, 48].We found that adding aspirin 12 h after rotavirus infection inhibited the expression of COX-2 in host cells, and after 24 h, compared with the RV infection group, the mRNA COX-2 expression increased, while the activity of PGE2 was statistically insignificant. Therefore, the inhibitory effect of aspirin on the rotavirus might not be through blocking COX mRNA expression and PGE2 activity. We will continue to in-depth and verify this result in the later stage of the experiment research. The results also showed that long-term aspirin administration lowered the expression levels of IL2 and IL10 in rotavirus infection rats.IL-10 was considered a master negative regulator of inflammation and a key component of cytokine system that regulated and suppressed the expression of proinflammatory cytokines during the recovery phases of infections and consequently reduced the damage caused by inflammatory cytokines [51, 52].Interleukin-2 (IL-2) was considered to be an essential growth factor for T cells and was implicated in initiating immune responses. The ability of IL-2 to act on various cell populations may promote inflammatory reactions changes in IL-2 sensitivity during virus infection [53]. Rotavirus-infected rats exhibited higher levels of IL-2 and IL-10, and the increased levels were partially reversed when aspirin was administered.

We also evaluated the effect of long-term aspirin administration on gut microbiota and the infectivity of rotavirus in rats. Most people who take aspirin for a long time are elderly patients. Therefore, rats continuously administered aspirin orally for 6 months were used to evaluate the infectivity of rotavirus infection. Although obvious diarrhea symptoms in rats had not been observed, virus replication still could be detected. The weight gain of rats was not affected by long-term aspirin administration and rotavirus infection. However, the abundance of gut microbiota in rats was changed significantly, and the rotavirus infection titer was also decreased compared with that of the group without aspirin.

In this study, the changes in gut microbiota in rats with aspirin treatment were analyzed by 16 S sequencing, and the results showed that the composition of gut microbiota in the NC group differed from that in the ASP groups. The main phylum represented in the gut microbiota are Bacteroidetes, Firmicutes, Actinobacteria, Proteobacteria, and Verrucomicrobia [37, 54]. This study found that after long-term aspirin administration, the expression of Firmicutes was significantly increased, the expression of Bacteroides was significantly decreased, and the proportion of Firmicutes/Bacteroides was also significantly increased. In addition, long-term aspirin administration was associated with significant changes in the relative abundance of many gut microbiota. Aspirin might be metabolized by microbial enzymes in the gut, and its metabolism could be affected by microbiota in the GI tract. However, the biotransformation of aspirin by gut microbiota has not yet been characterized. Probably, gut microbiota are involved in aspirin metabolism and may be able to modulate the inhibition of rotavirus efficacy of aspirin by altering the gut microbiota [55]. Changes in the microbiota can confer resistance or promote infection by pathogenic bacteria. Pathogenic bacteria exploit microbiota-derived sources of carbon and nitrogen as nutrients and regulatory signals to promote their growth and virulence [56].

We speculate that long-term oral aspirin affects the composition of gut microbiota in rats and leads to changes in the ability of rats to infect rotavirus. This effect is probably related to the different hosts, different titers of virus infection, as well as the duration of infection. However, this conjecture needs to be further studied in subsequent experiments. These results indicate that the rotavirus infection in the intestines of elderly patients who take aspirin for a long time may be suppressed. However, the change in their gut microbiota may lead to functional disorders of the intestines.

In conclusion, aspirin effectively inhibits the infection and replication of rotavirus in vitro and in vivo. The mechanism by which aspirin reduces rotavirus infection may involve the inhibition of rotavirus-induced expression of COX-2, IL-2 and/or IL-10. Long-term oral aspirin results in the alteration of gut microbiota composition, which can underlie its protective effects.

### Electronic supplementary material

Below is the link to the electronic supplementary material.

**Supplementary Figure 1**. In vitro cytotoxicity of aspirin. The in vitro cytotoxicity of aspirin was assessed on physiologically active cells: MA104 (A), Caco-2 (B), and CV-1 (C). The titration curves show the dose-dependent cytotoxicity determined using three replicates for each serial dilution at 12 h (black), 24 h (red), 36 h (blue) and 48 h (green) for each of the cell lines.

**Supplementary Figure 2.** Fluorescent cells could not be detected in the ASP group at any stage of the 30-day time course.The viral titer and virus RNA were evaluated on 6, 12, 18, 24, 30 days in ASP-RV group by fluorescent assay. The ASP-RV group was not detected fluorescent.

**Supplementary Figure 3.** Schedule methodology of aspirin and virus into rat model
