# Supplementary figures and images for: Aspirin inhibits rotavirus replication and alters rat gut microbial composition
Source: Virol J. 2023 Oct 17;20:237. doi: 10.1186/s12985-023-02199-5 (PMC10580602; doi:10.1186/s12985-023-02199-5)

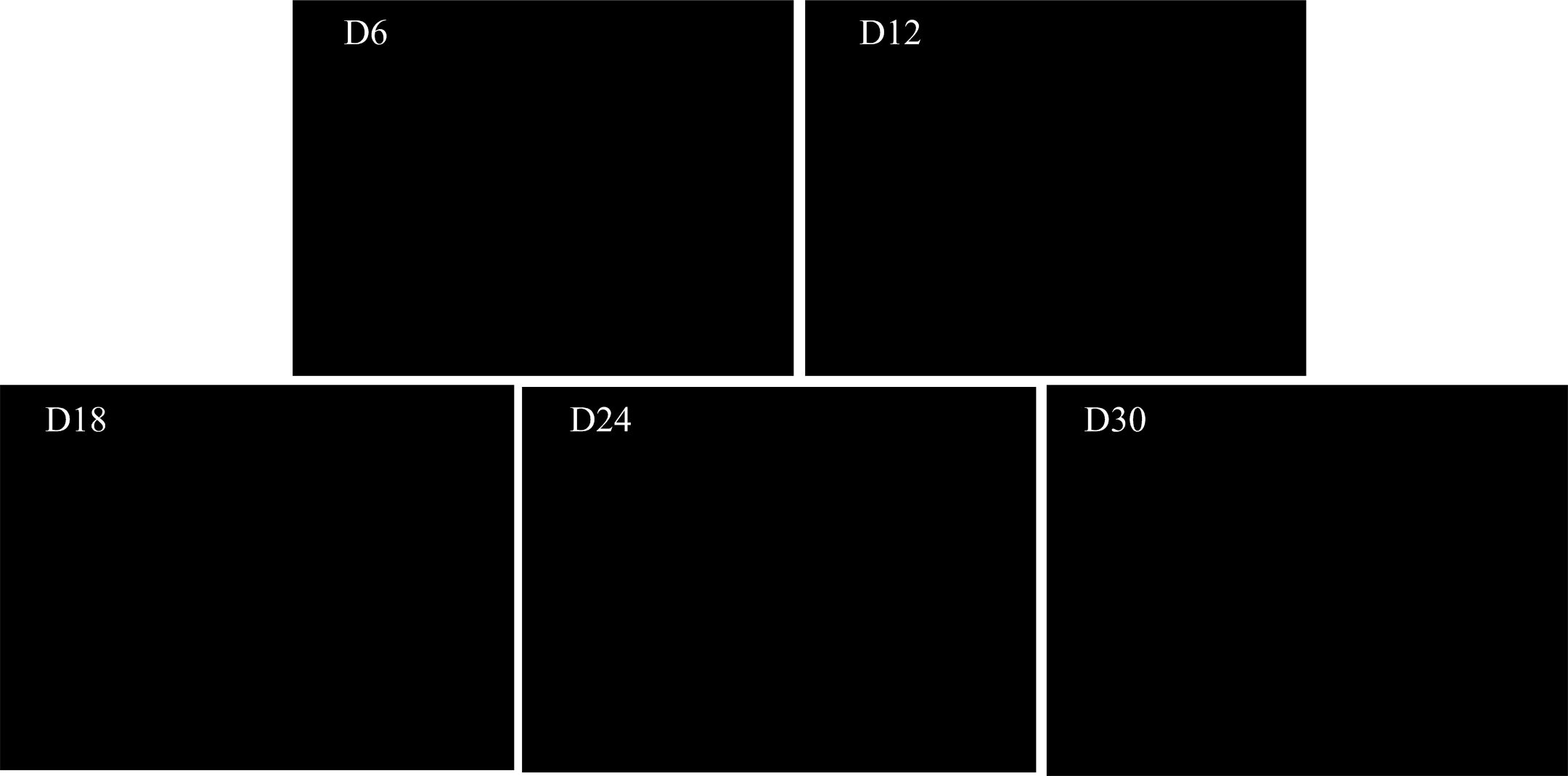

Supplement: Supplementary file 1 — Supplementary Figure 1. In vitro cytotoxicity of aspirin. The in vitro cytotoxicity of aspirin was assessed on physiologically active cells: MA104 (A), Caco-2 (B), and CV-1 (C). The titration curves show the dose-dependent cytotoxicity determined using three replicates for each serial dilution at 12 h (black), 24 h (red), 36 h (blue) and 48 h (green) for each of the cell lines. [file 12985_2023_2199_MOESM1_ESM.png]

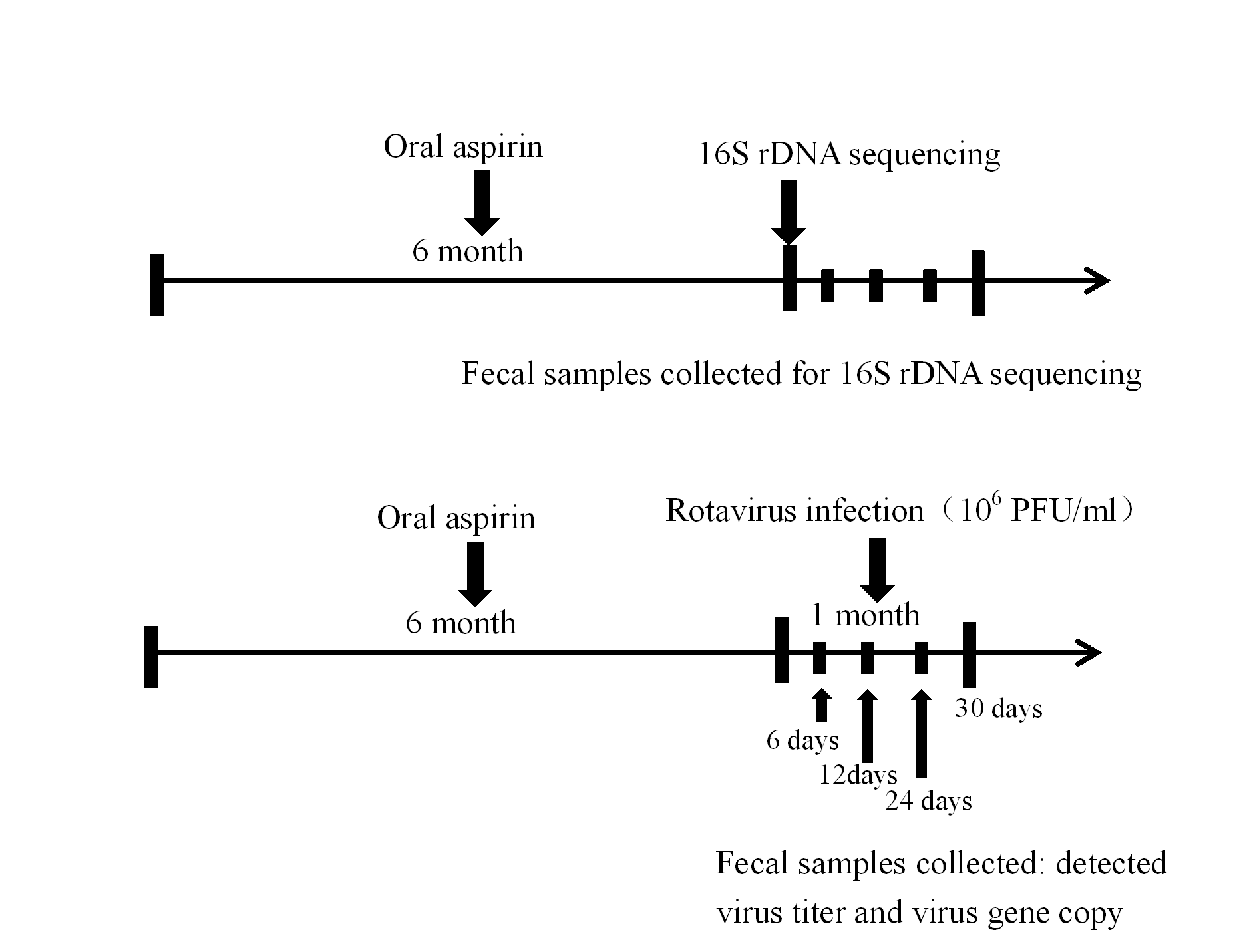

Supplement: Supplementary file 2 — Supplementary Figure 2. Fluorescent cells could not be detected in the ASP group at any stage of the 30-day time course.The viral titer and virus RNA were evaluated on 6, 12, 18, 24, 30 days in ASP-RV group by fluorescent assay. The ASP-RV group was not detected fluorescent. [file 12985_2023_2199_MOESM2_ESM.png]

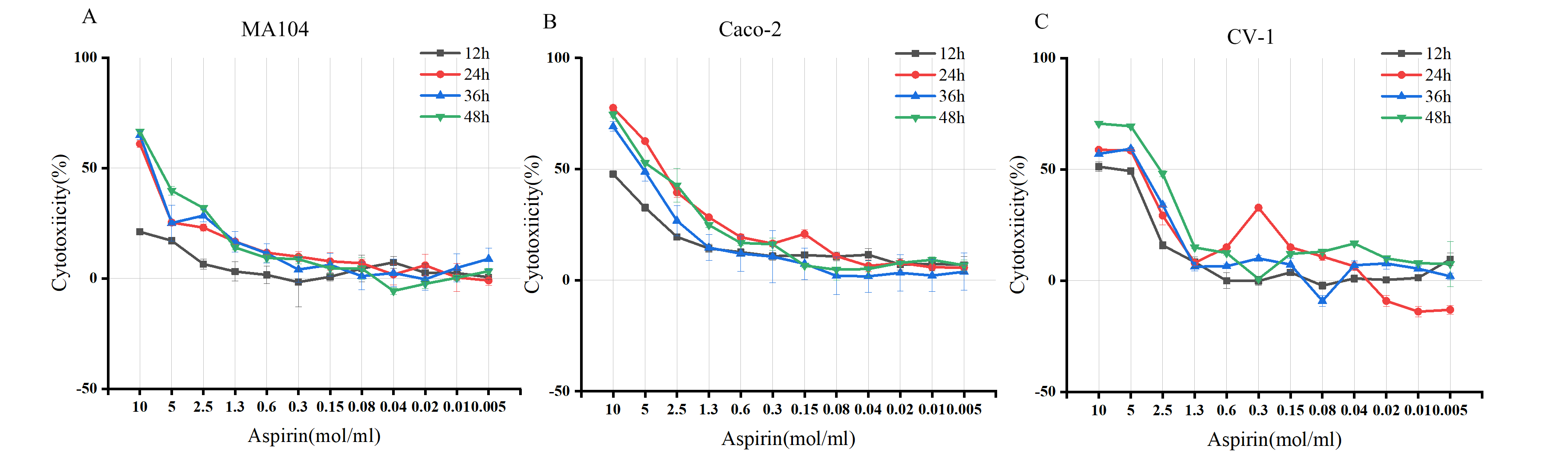

Supplement: Supplementary file 3 — Supplementary Figure 3. Schedule methodology of aspirin and virus into rat model [file 12985_2023_2199_MOESM3_ESM.png]
